# Microeukaryotic and Prokaryotic Diversity of Anchialine Caves from Eastern Adriatic Sea Islands

**DOI:** 10.1007/s00248-021-01760-5

**Published:** 2021-04-26

**Authors:** Katarina Kajan, Neven Cukrov, Nuša Cukrov, Renée Bishop-Pierce, Sandi Orlić

**Affiliations:** 1 Division of Materials Chemistry, Ruđer Bošković Institute, Zagreb, Croatia; 2 Center of Excellence for Science and Technology-Integration of Mediterranean Region (STIM), Zagreb, Croatia; 3 Division for Marine and Environmental Research, Ruđer Bošković Institute, Zagreb, Croatia; 4 Pennsylvania State University, Dunmore, PA USA

**Keywords:** Anchialine cave, Salinity gradient, Microeukaryotic plankton, Prokaryotic plankton, Diversity

## Abstract

Anchialine ecosystems in the eastern Adriatic Sea are diverse both morphologically and biologically. In this study, for the first time, we explored the microeukaryotic and prokaryotic community of anchialine caves in the Mediterranean region using high-throughput sequencing. Four anchialine caves located on nearby islands with a well-pronounced salinity gradient were sampled at the surface freshwater area, halocline area, and seawater area. Sequencing revealed a surprisingly wide diversity of the microeukaryotic and prokaryotic community with the relative abundance of major phyla differing within the salinity gradient and between the caves. Interestingly, microeukaryotic and prokaryotic communities clustered into four groups based on location, pointing out that sampled anchialine caves have different microbial community patterns and high microbial endemism. Our results indicate that even with the halocline acting as a selecting barrier, the salinity is not the only community structuring factor. Despite the short geographical distance, the isolation of anchialine caves facilitated high microbial community adaptation and endemism. Our study suggests that anchialine caves represent reservoirs of new biodiversity, maintaining unique and complex microbial diversity influenced by biotic interactions and abiotic environmental conditions.

**Supplementary Information:**

The online version contains supplementary material available at 10.1007/s00248-021-01760-5.

## Introduction

Aquatic microbial communities are affected by many environmental factors that determine their diversity and abundance [1, 2]. Changing their community structure patterns across space and time, microbes occupy broad ecological niches. Facilitating the application of amplicon sequencing methods and computational analysis has expanded the scope of microbial community profiling on previously unexplored extreme and complex habitats, ranging from caves to deep-sea hydrothermal vents [3, 4].

Anchialine ecosystems are defined as “tidally-influenced subterranean estuaries within crevicular and cavernous karst and volcanic terrains, that extend inland to the limit of seawater penetration” [5]. Due to the sea and groundwater connections, they possess both seawater and freshwater influences [6]. Although they have a worldwide distribution, habitats fitting this ecosystem definition are considered relatively rare, located in tropical and moderately warm climatic zones [7]. Anchialine ecosystem habitats represent an important long-term reservoir of species diversity and endemism maintained by limiting resources of light, nutrients, and oxygen. Geographic isolation, together with abiotic pressures, such as halocline, chemocline, and oxycline, are acknowledged promoters of evolution in organisms in these habitats [8]. Defined clines in anchialine ecosystems act as a selecting barrier affecting species distribution making them as exquisite models for species diversity research [9]. Although parameters such as temperature, light resource, and nutrient limitation remain relatively stable, these factors may differ considerably between and within the anchialine ecosystem [10, 11].

Thus far, we have begun to understand better the importance and function of microorganisms and how microbial diversity is distributed across environments, yet the microbial community of anchialine ecosystems is still poorly investigated compared to other aquatic environments. Most anchialine cave studies documented endemism among eukaryotes [12]. Relatively few studies have attempted to record the full diversity of microbial communities in anchialine ecosystems [13–15], even though these studies resulted in descriptions of new species using novel molecular tools. In the region of the eastern Adriatic Sea, the majority of anchialine ecological studies have been based on the taxonomic research of stygobiotic metazoans [16], the distribution of trace metals [17], and iodine species and nutrients [18]. Technical difficulties in sampling anchialine ecosystems limit the ability to study microbial communities. These environments along the Adriatic coast are spatially complex habitats, accessible only by speleologists and scuba divers. Anchialine caves represent a unique and understudied environment common in the area of the eastern Adriatic coast [19].

To our knowledge, this study presents the first investigation of the microeukaryotic and prokaryotic plankton community across the halocline of anchialine caves in the Mediterranean region using amplicon sequencing. The primary objective was the identification of abiotic factors that are of importance for structuring microbial communities in four anchialine caves at three depths defined by sharp vertical salinity stratification. We hypothesized that microeukaryotic and prokaryotic communities in anchialine caves have a similar habitat pattern shifting through the halocline area and have high variations in diversity between the sampling depths.

## Materials and Methods

### Site Description and Sample Collection

**Fig. 1:**
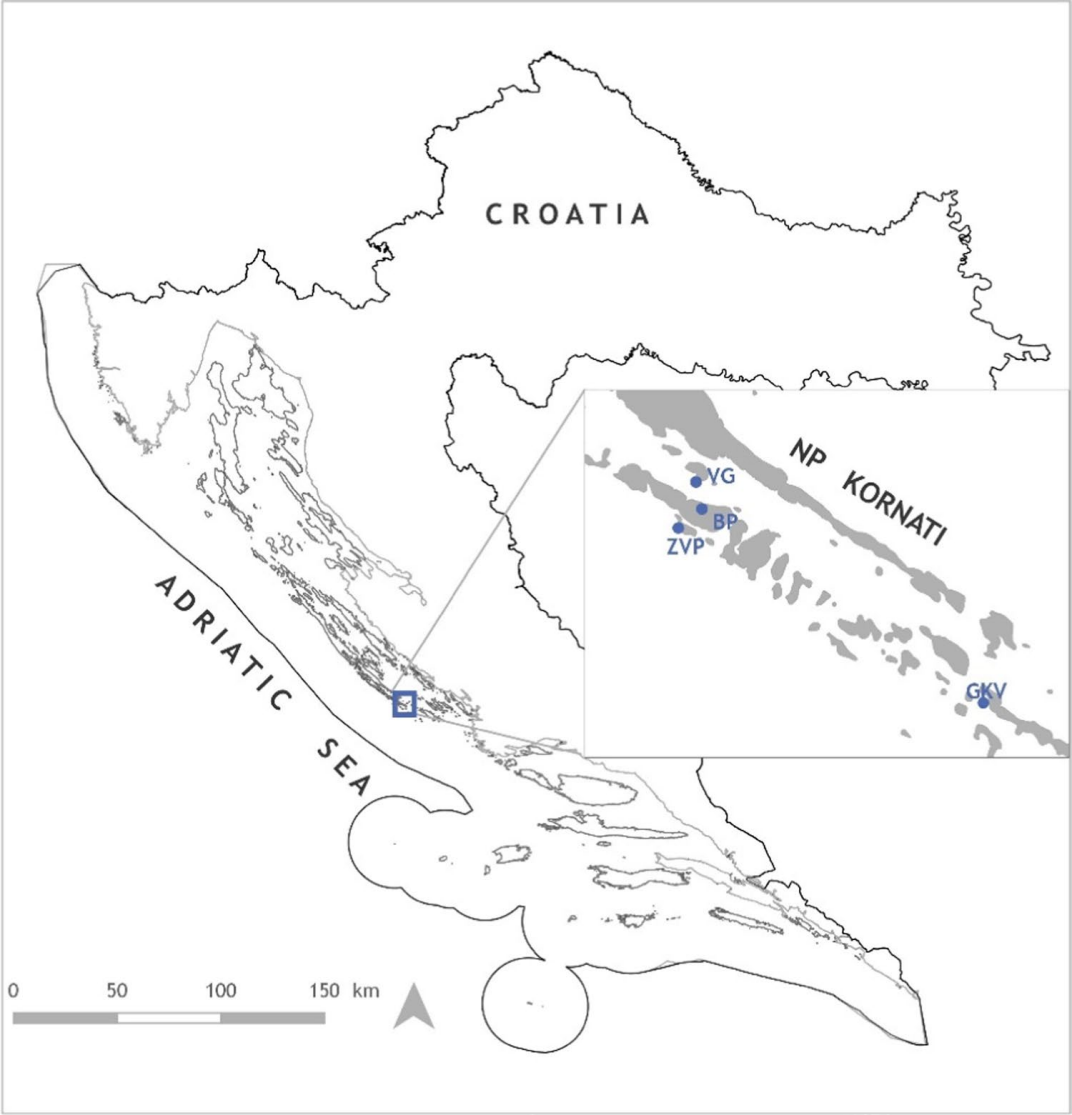
Location of anchialine caves on the islands of National Park Kornati. Vjetruša cave (VG), Blitvica cave (BP), Gravrnjača cave (GKV), and Živa voda cave (ZVP)

Sampling took place in National Park Kornati, situated in the eastern part of the Adriatic Sea, Croatia (Fig. 1). Four anchialine caves located on different islands, Vjetruša (VG), Blitvica (BP), Živa Voda (ZVP), and Gravrnjača (GKV), were sampled in June 2016 during the expedition of the Croatian Biospeleological Society members (Table 1). Sampling depths were established based on the vertical salinity gradient. Three water samples were taken in each cave: sample in the area of fresh to brackish surface water (above the halocline), sample within the halocline, and the area of seawater (below the halocline). Water samples for molecular and chemical analysis were collected in bottles with a total of 2 L from progressively increasing depths, ensuring that each sample was of undisturbed water [20].

**Table 1:** Details of the sampled anchialine caves

	Vjetruša (island Guštac; VG)	Blitvica (island Piškera; BP)	Gravrnjača (island Kurba Vela; GKV)	Živa Voda (island Panitula Vela; ZVP)
Location	43° 46′ 27.2″ N 15° 20′ 59.3″ E	43° 45′ 54.8″ N 15° 21′ 09.5″ E	43° 42′ 16.4″ N 15° 28′ 23.0″ E	43° 45′ 40.54″ N 15° 20′ 29.65″ E
Distance from the coast (m)	120	100	30	50
Cave depth (m)	60	70	40	10
Water depth (m)	24	50	31	6

### Physical and Chemical Analysis

Salinity stratification in the anchialine water column was determined by instant CTD probe (EXO2, YSI, USA), taken at each station before all other samples, to characterize the halocline. Physical parameters (salinity, pH, dissolved oxygen (DO), and water temperature) were measured with depth in situ using diver-carried multiparameter data loggers Hach HQ40D Portable Multi-Parameter Meter (Hach Company, Loveland, CO, USA). Concentrations of total nitrogen (TN), ammonium with organic nitrogen (N-NH_4_^+^  + N_org_), nitrogen-nitrite (N-NO_2_^−^), nitrogen-nitrate (N-NO_3_^−^), orthophosphate (PO_4_^3−^), and total dissolved carbon (TOC) were determined in collected water samples (300 mL). The concentrations of nutrients were measured on a Perkin Elmer Lambda 25 UV/Vis spectrometer. Nitrate, nitrite, and ammonium were measured according to Zhang and Fischer [21], with method detection limits of 0.5 μmol L^−1^, 0.03 μmol L^−1^, and 0.4 μmol L^−1^, respectively, with overall precision ± 10%. Orthophosphate concentrations were analyzed according to ISO 6878:1998(E). The method is based on the formation of the phosphomolybdate complex, which is subsequently reduced with ascorbic acid to form a strongly colored blue molybdenum complex; the absorbance is measured spectrometrically at 880 nm. Detection limits in orthophosphate analyses were 0.04 μmol L^−1^ and 0.15 μmol L^−1^, respectively, while precision was typically better than ± 10%.

A sample aliquot was filtered on 25-mm glass filters (GFF, Whatman) using an all-glass filtering system (Wheaton) under vacuum to determine the total organic carbon content. All glass equipment (filter, tubes, filtering system) was calcined at 450 °C for 4 h before use. The resulting dissolved fraction was stored in a 24-mL glass tube equipped with a Teflon/silicone septum (Wheaton), poisoned with 50 μL of 1 mol L^−1^ NaN_3_ (Aldrich), and stored in the dark at 4 °C until analysis. Filters were dried to constant weight at 60 °C and then exposed to HCl fumes for 4 h to remove all inorganic carbon [22]. The dissolved organic carbon (DOC) concentrations were determined using a Shimadzu TOC-VCSH analyzer and the high-temperature (680 °C) catalytic oxidation method with IR detection of CO_2_ [23], calibrated using potassium hydrogen phthalate (Fisher Scientific, Analytical Reagent grade) [24]. The particulate organic carbon (POC) concentration on the filters was determined using the same equipment via the Shimadzu SSM-5000 module, which uses catalytic oxidation at 950 °C, and is calibrated using glucose (Fisher Scientific, Analytical Reagent grade). The sum of the DOC and the POC yielded the total organic carbon (TOC) content to 10% accuracy. Precision was typically better than ± 5%.

### DNA Extraction, Amplification, and Sequencing

Water samples (1 L) were filtered on 0.2-µm pore size polycarbonate filters. According to the manufacturer’s guidelines, total genomic DNA was extracted from filters with the DNeasy PowerWater Kit (Qiagen GmbH Hilden, Germany). The hypervariable V9 region of the eukaryotic SSU rRNA gene was amplified using the primer pair 1391F (5′-GTACACACCGCCCGTC-3′) and EukB (5′-TGATCCTTCTGCAGGTTCACCTAC-3′) following the protocol of Stoeck et al. [25]. For the bacterial dataset, the hypervariable V4 region of the 16S rRNA gene was amplified using primer pair 515F (5′-GTGCCAGCMGCCGCGGTAA-3′) and 806R (5′-GGACTACHVHHHTWTCTAAT-3′) [26]. To minimize PCR bias, three individual reactions per sample were prepared and pooled prior to sequencing. Paired-end sequencing of purified 18S V9 amplicons was conducted on an Illumina NextSeq platform generating 150-bp reads (SeqIT GmbH & Co. KG, Kaiserslautern, Germany). The bacterial reads were sequenced on an Illumina MiSeq platform generating 250-bp paired-end reads (MR DNA, Molecular Research LP, Shallowater, TX, USA). Raw demultiplexed reads were deposited at the ENA’s Sequence Read Archive and are publicly available under project number PRJEB43761.

### Sequence Processing and Analysis

Paired-end reads were quality trimmed using the bbduk function and merged using bbmerge function of the BBMap package (v. 38.71; https://sourceforge.net/projects/bbmap/) and quality-filtered using the split_libraries.py script implemented in QIIME v. 1.8.0 to remove low-quality reads [27]. Only reads with exact barcodes and primers, unambiguous nucleotides, and a minimum length of 90 (18S V9 region) and 250 (16S V4 region) base pairs were retained. Chimeric sequences (representing sequencing artifacts) were identified and removed using UCHIME [28]. Non-chimeric reads were clustered into Operational Taxonomic Units (OTUs) with SWARM v. 3.0.0 [29] using *d* = 1, clustering amplicons by using a local clustering threshold. For the microeukaryotic dataset, the taxonomic assignment was done using *blastn* in BLAST v. 2.9.0 [30] against the NCBI nucleotide database. Prokaryotic OTUs were blasted against the SILVA database (SILVA release 132; December 13, 2017). Non-target OTUs (metazoans, embryophytes in the microeukaryotic dataset; chloroplasts in the prokaryotic dataset), as well as singletons and doubletons, were excluded. Resulting OTUs were filtered by the quality of the blast results (≥ 98% identity). To minimize biases associated with sequencing and allow comparison between the samples, standardization among samples was performed by randomly subsampling the table of OTUs to the minimum read level using the *rrarefy* function of the R package “vegan” [31]. The resulting files were used as a basis for further statistical analyses.

All statistical analyses were performed in the R environment (v. 4.0.4) [32] and visualized using the “ggplot2” package [33]. The alpha diversity was estimated as the OTU richness, Shannon–Wiener and Simpson index for each microbial community using the “vegan” package. Shared and unique OTUs of microeukaryotic and prokaryotic community were distinguished through a Venn diagram (package “VennDiagram” [34]). Prior to beta diversity analysis, Hellinger transformation was applied to datasets of microbial communities. The similarity of the microeukaryotic and prokaryotic community between the anchialine caves and the sampling depths were tested by principal coordinate analysis (PCoA) based on Bray–Curtis dissimilarity distance (package “ape” [35]). Permutational multivariate analysis of variance (PERMANOVA) was used to test whether the partitioning of microbial communities was affected significantly by the anchialine cave or the sampling depth (package “vegan”). The function *envfit* of the package “vegan” was applied to the results of PCoA to evaluate the correlations with environmental factors and the significance of this regression by permutations test. Co-inertia analysis (CIA) based on the PCoA results was used to evaluate the correlation of microbial communities using the “ade4” package [36]. CIA results were tested by the Monte Carlo test to evaluate significance. A simplified version of the R script is available online (https://github.com/kkajan/anchialine-miceco).

## Results

### Environmental Characteristics of Anchialine Water Columns

**Fig. 2:**
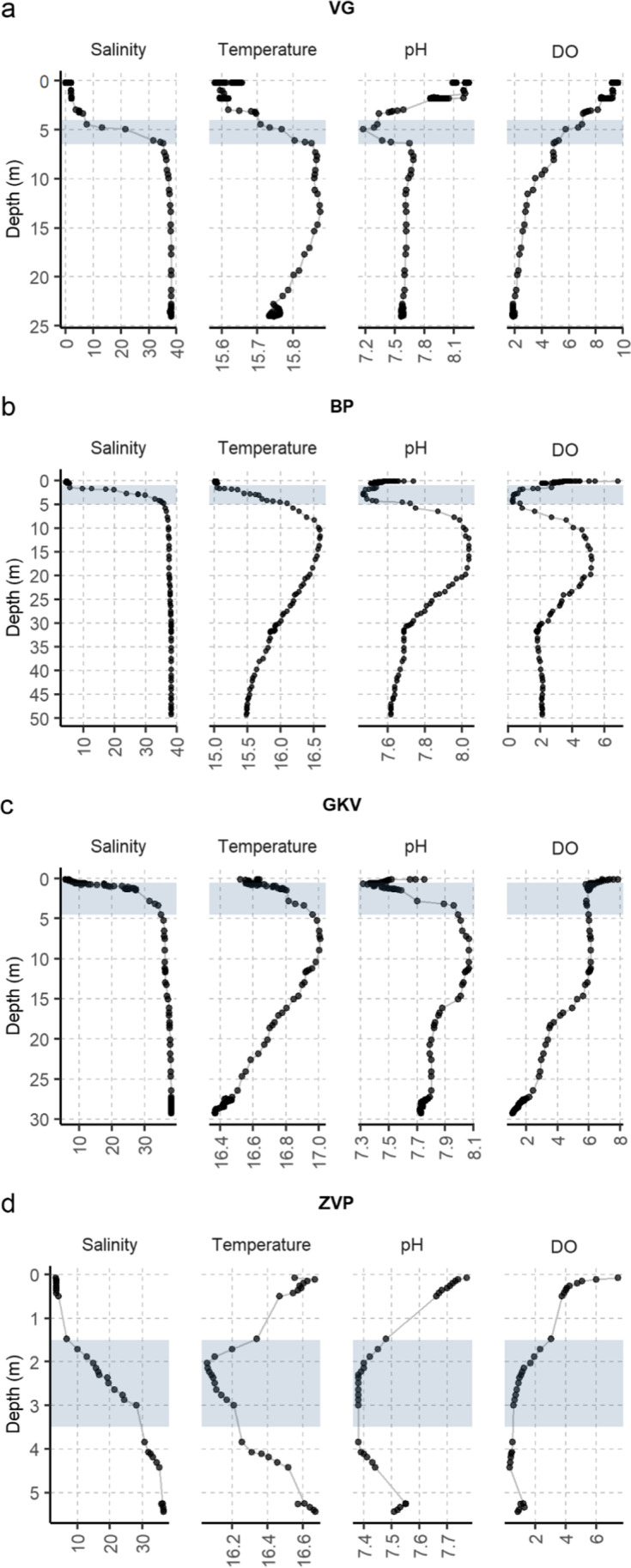
Hydrographical profile in a depth profile of anchialine caves in June 2016. **a** Vjetruša cave (VG), **b** Blitvica cave (BP), **c** Gravrnjača cave (GKV), and **d** Živa voda cave (ZVP). From left to right: salinity (‰), temperature (°C), pH, and dissolved oxygen (DO; mg L^−1^). Blue-colored rectangles highlight the halocline area

The water in all caves during the sampling was heavily stratified due to a strong salinity gradient (Fig. 2). Salinity varied from an average of 3.98‰ at the surface (min. 1.88‰ (VG), 5.93 ‰ max. (GKV)) and 37.87‰ in the bottom layer of the caves (min. 36.65‰ (ZVP), 38.34 ‰ max. (BP)). A well-defined halocline was detected in all caves at a depth of approximately 3 m (min. 2.2 m (ZVP), max. 3.8 m (BP)). In contrast to salinity, temperature and pH varied in smaller intervals (16.03 ± 0.53 °C; pH 7.77 ± 0.22). In VG and ZVP caves, pH and temperature decreased with depth, while in BP and GKV, the highest pH values were recorded directly below the halocline. DO steadily decreased with depth from normoxic to hypoxic condition in all caves except BP. A decrease of DO was recorded in the halocline area of BP cave with a subsequent rapid increase below the halocline, reaching a maximum at ~ 16 m (0.29–5.21 mg L^−1^). The highest TN at the surface was measured in VG cave (7 mg L^−1^) and the lowest in BP (0.39 mg L^−1^) (Table S1). The lowest TOC amounts were detected below the halocline in all caves (0.56 ± 0.21 mg L^−1^).

### Taxonomic Composition and Diversity of the Microeukaryotic Community

Illumina sequencing of three depths within the four anchialine caves resulted in a total of 31,045,886 V9 SSU rRNA reads, of which 3,931,978 reads were assigned to 2,991 target microeukaryotic OTUs. The majority of clean reads were not assigned (~ 27%) or were assigned to the non-target OTUs (metazoans, embryophytes, and Bacteria; ~ 54%). The total number of assigned reads ranged between 57,334 (BP1, above halocline) and 979,181 (GKV2, halocline). Taxonomic richness varied notably along the salinity gradient with the lowest number of OTUs detected below the halocline in cave VG (*n* = 463) and the greatest richness above the halocline in BP cave (*n* = 1,093) followed by the richness of the halocline in ZVP (*n* = 914) (Fig. S1a). The highest microeukaryotic diversity, according to the Shannon–Wiener index, was recorded in the area of the halocline in BP cave (4.04) and VG cave (4.02), while the lowest was in the halocline of GKV cave (1.57).

**Fig. 3:**
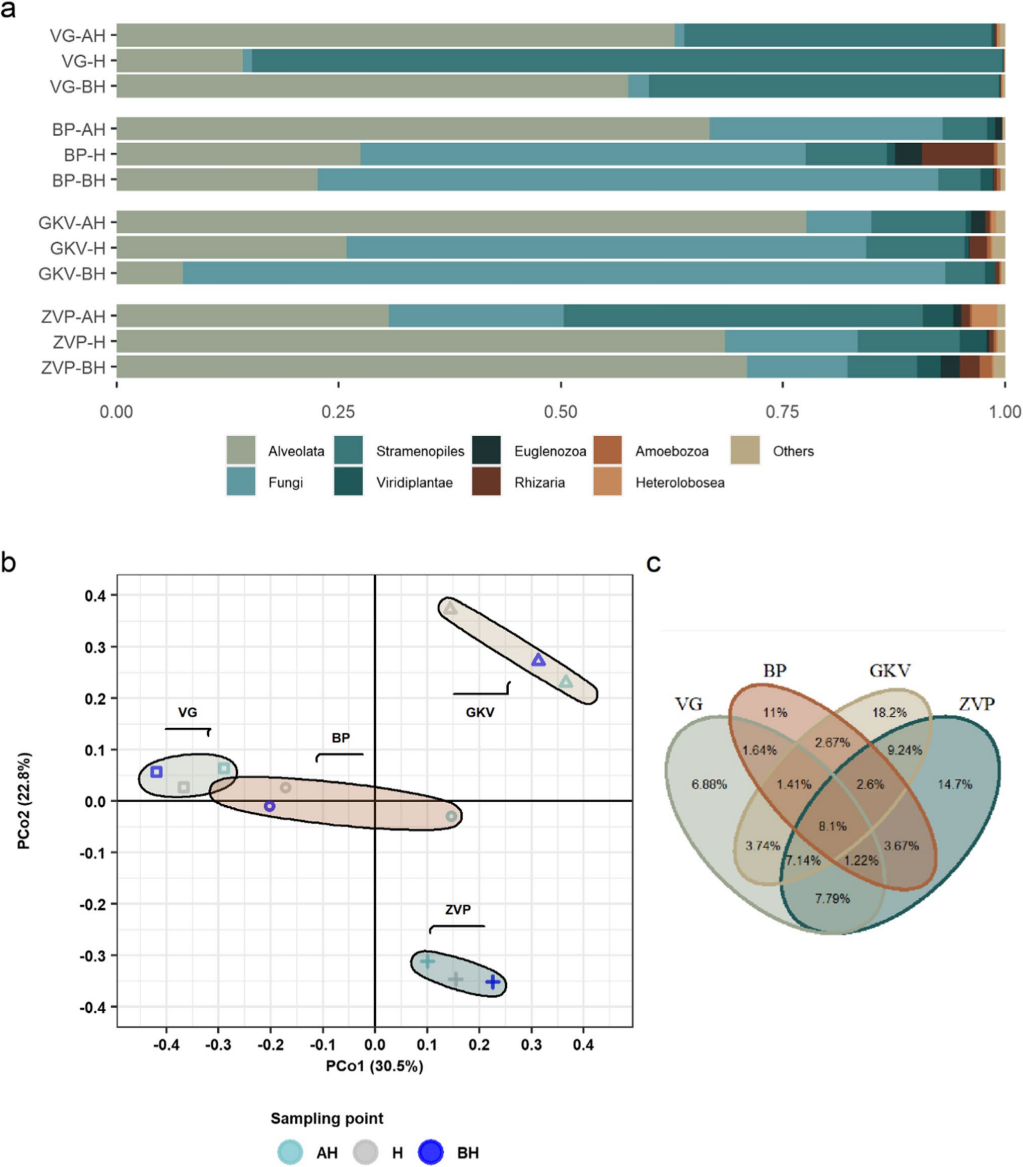
**a** Taxonomic composition of the microeukaryotic community in anchialine caves based on the relative abundance of the most abundant assigned higher taxonomic rank (≥ 0.01). Higher taxonomic ranks with relative abundance < 0.01 were aggregated into “others.” **b** Principal coordinate analysis (PCoA) of microeukaryotic community in anchialine caves using Bray–Curtis distances on the level of OTUs. The plot is color-coded by the sampling point (above halocline (AH), halocline (H), and below halocline (BH)) and shape-coded by the anchialine cave. Groups are color-coded by the sample origin. **c** Venn diagram showing the percentage of microeukaryotic OTUs overlap between the anchialine caves

**Fig. 4:**
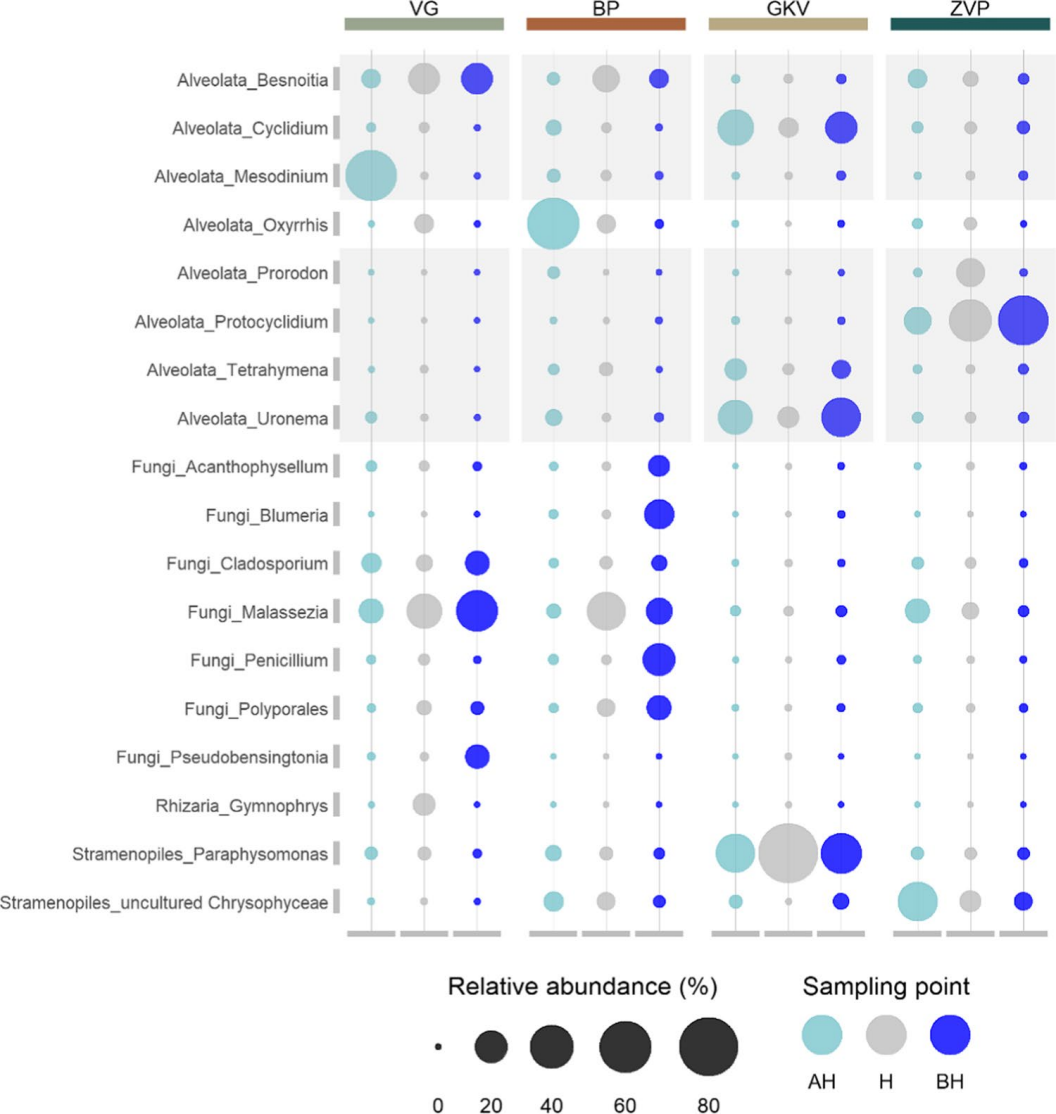
Microeukaryotic community at the genus level with the relative abundance ≥ 5% in at least one sample. The bubble size represents the relative abundance of the genera and the color represents the sampling point (above halocline (AH), halocline (H), and below halocline (BH)). Gray-colored rectangles highlight the genera of Ciliophora

Altogether, 22 higher taxonomical levels were recorded wherein an average microeukaryotic community of anchialine caves consisted of Alveolata (44.37%), Fungi (29.02%), Stramenopiles (21.94%), Rhizaria (1.29%), and Viridiplantae (1.26%) (Fig. 3a). On average, the ZVP cave was dominated by 56.7% of Alveolata reads, of which the majority were affiliated with Ciliophora (45.62%). An average of 20% of the reads belonged to Stramenopiles, with Chrysophyceae as the dominant group (17.3% of the reads; the highest above the halocline with 35.42% of reads). Fungi were recorded in 15.3% of average reads in the ZVP cave, with the highest contribution of Dikarya (19.6%) in the marine-like sample. In contrast to the shallowest sampled cave, GKV cave was dominated by Stramenopiles (52.8%) and Alveolata (44.9%). A high number of Chrysophyceae reads were found in the halocline of GKV (83.5%), with Chromulinales as the main lineage. The shift of Alveolata and Fungi was recorded in the salinity gradient of BP cave, with the domination of Alveolata reads in the surface area (77.6%; Ciliophora (12.2%) and Dinophyceae (64.1%)) and with Fungi reads in the marine-like area (85.8%; Dikarya (84.7%)). A similar composition to BP cave was identified in VG cave with Alveolata dominating above the halocline (66.7%; Ciliophora (60.6%) and Fungi dominating below the halocline (69.9%; Dikarya (69.1%)). The average reads of Rhizaria and Viridiplantae were relatively low, with 1.3%. Based on the level of genera, microeukaryotes with relative abundance ≥ 5% belonged to Stramenopiles, Rhizaria, Fungi, and Alveolata (Fig. 4). The Venn diagram showed the overlap between the anchialine caves with a total of 8.1% shared target microeukaryotic OTUs (Fig. 3c).

### Taxonomic Composition and Diversity of the Prokaryotic Community

Sequencing of V4 SSU rRNA resulted in a total of 1,992,407 reads of which 1,271,717 reads were clustered and classified into 12,088 target prokaryotic OTUs. The lowest number of target reads was recorded above the halocline in BP cave (*n* = 36,528) followed by the sample below the halocline of VG cave (*n* = 46,722). In contrast to BP cave, the sample above the halocline in GKV cave was presented by the highest number of reads (*n* = 222,403), while below, the halocline with the highest number of OTUs (*n* = 2,974). The prokaryotic community richness showed a similar pattern in both the VG and BP cave with the highest richness recorded in the halocline, while in the ZVP cave, the greatest richness was recorded below the halocline (*n* = 3,033; Fig. S1b). Shannon–Wiener index showed the highest diversity in samples below the halocline in caves GKV (5.7) and BP (5.9), while in cave VG, the highest diversity was recorded in halocline (5.3).

**Fig. 5:**
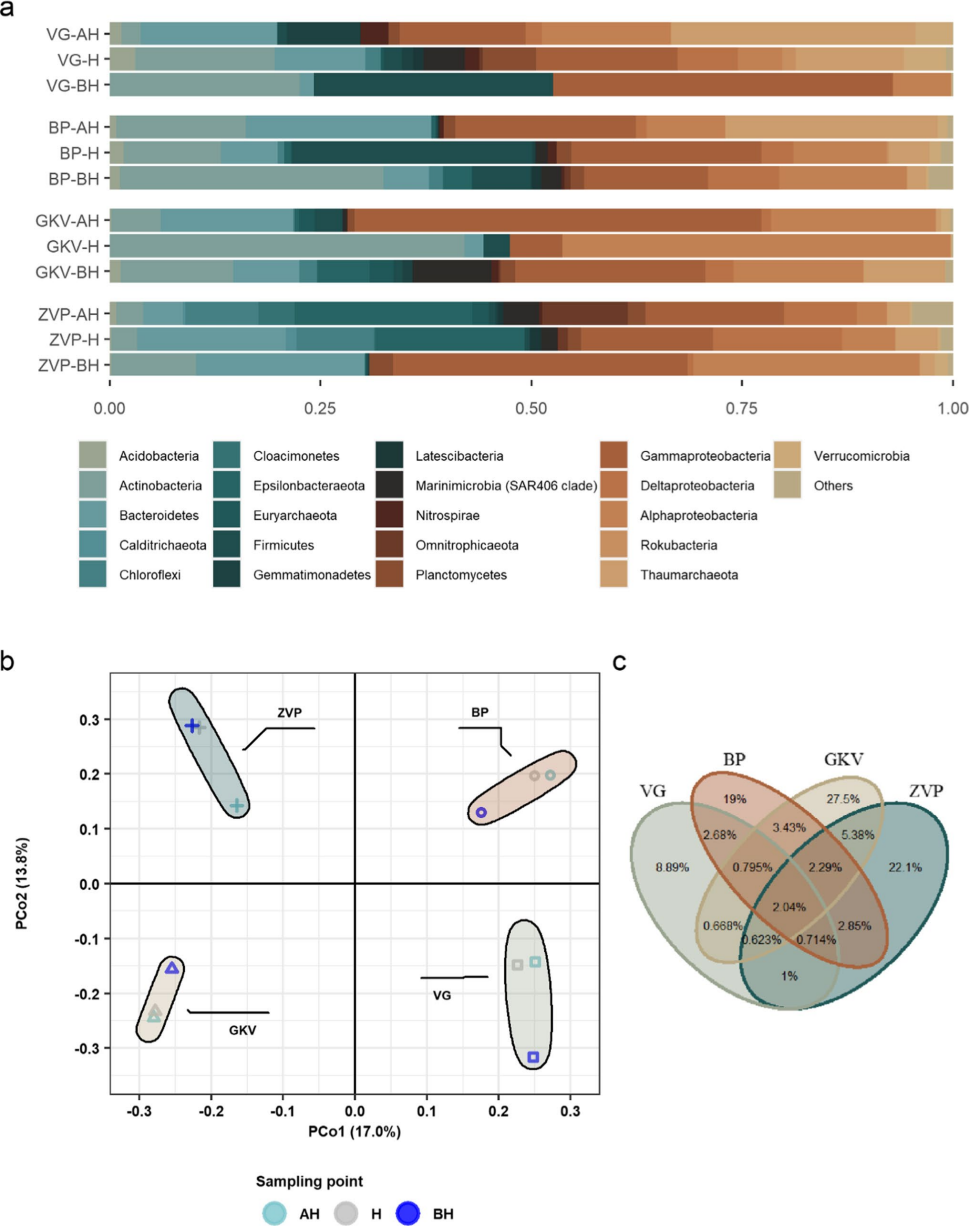
**a** Taxonomic composition of the prokaryotic community in anchialine caves based on the relative abundance of the most abundant phylum or classes (for Proteobacteria) (≥ 0.01). Phyla with relative abundance < 0.01 were aggregated into the group reported as “others.” **b** Principal coordinate analysis (PCoA) of prokaryotic community in anchialine caves using Bray–Curtis distances on the level of OTUs. The plot is color-coded by the sampling point (above halocline (AH), halocline (H), and below halocline (BH)) and shape-coded by the anchialine cave. Groups are color-coded by the sample origin. **c** Venn diagram showing the percentage of prokaryotic OTUs overlap between the anchialine caves

**Fig. 6:**
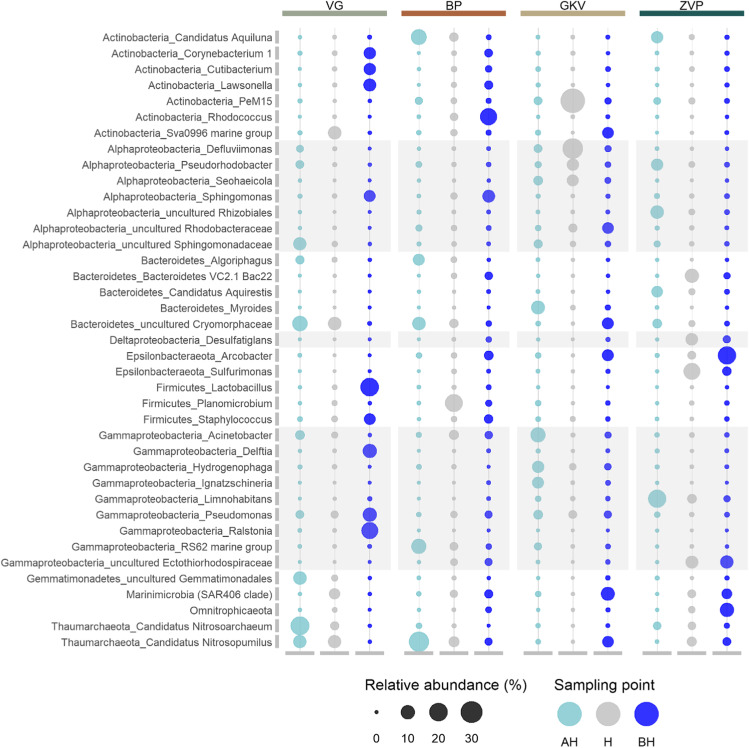
Prokaryotic community at the genus level with the relative abundance ≥ 5% in at least one sample. The bubble size represents the relative abundance of the genera and the color represents the sampling point (above halocline (AH), halocline (H), and below halocline (BH)). Gray-colored rectangles highlight the genera of Proteobacteria

Altogether, 68 prokaryotic phyla were detected, from which nine were archaeal (Fig. 5a). The most abundant prokaryotes in all four caves were affiliated with the phyla Proteobacteria, with the highest average of Gammaproteobacteria (22.9%), followed by Alphaproteobacteria (15.1%) and Deltaproteobacteria (4.3%). VG, BP, and ZVP cave in average were dominated by Gammaproteobacteria (24%, 19.5%, 22.4%), respectively, while GKV cave was dominated by Alphaproteobacteria (27.8%) followed by Gammaproteobacteria (25.7%). Archaea were numerous in cave VG and BP in the surface area above the halocline with Thaumarchaeota (29%, 25.2%) of which in total Nitrososphaeria contributed (29%, 25.2%). The bacterial community of the VG also consisted of Bacteroidetes and Gemmatimonades with the contribution above and in the area of the halocline, and Actinobacteria and Firmicutes with the highest contribution below the halocline (22.4%, 28.3%). Actinobacteria were also present, with the highest amount below the halocline in BP (22.5%) and at the halocline in the GKV (42%). A higher amount of Epsilonbacteraeota was recorded in the ZVP cave in the halocline area and below (17.7%, 21.1%), while Bacteroides were recorded above and in the area of the halocline (20%, 17.6%). The halocline in GKV cave was dominated by Actinobacteria (42%) with the clade PeM15 (39.1%). Based on the genus level, the prokaryotic community was dominant with the highest contribution of Gammaproteobacteria, Bacteroidetes, Alphaproteobacteria, and Actinobacteria (Fig. 6). The prokaryotic community shared only 2% of the target prokaryotic OTUs between caves, showing a high contribution of unique OTUs in BP, GKV, and ZVP, respectively (Fig. 5c).

### Comparison of Microbial Communities

**Fig. 7:**
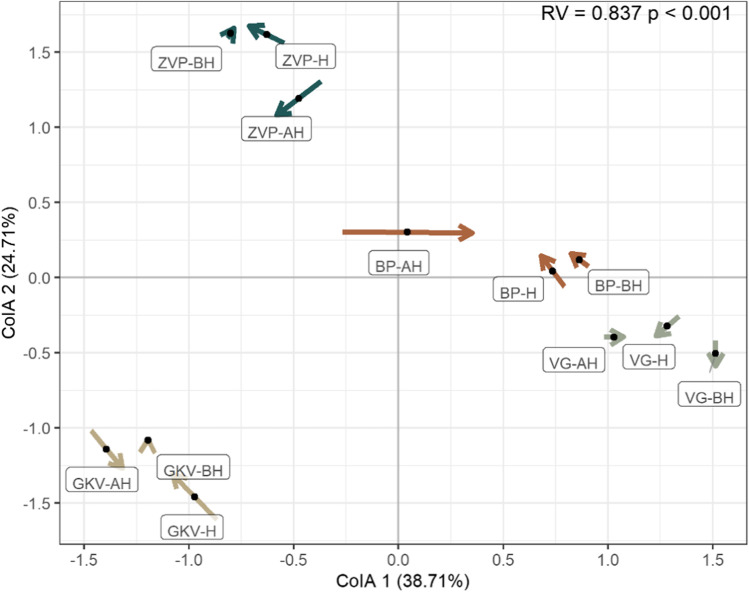
Comparison of the microeukaryotic and prokaryotic community diversity based on co-inertia analysis (CIA). The beginning of the arrow presents the theoretical position of the microeukaryotic sample and the end of the arrow presents the prokaryotic sample. Arrows are color-coded by the anchialine cave indicating two connected projections whereby the length of the line indicates the divergence between the two datasets. Significant RV value (*P* < 0.001) is marked on the plot. Sample name abbreviations refer to the area of a sampling point: above halocline (AH), in the halocline (H), and below halocline (BH)

The PCoA plot revealed the differences between the anchialine caves within the microeukaryotic and prokaryotic community generally clustering samples in line with their cave origin (Figs. 3b and 5b). This result was confirmed by two-way PERMANOVA analysis, showing that both the microeukaryotic and prokaryotic community of the anchialine caves differed significantly from each other (*P* < 0.001). The communities were only affected by temperature, while no correlation was observed with other measured environmental parameters on the OTU level. CIA resulted in a high significant correlation of microeukaryotic and prokaryotic communities of anchialine caves (RV = 0.8369, *P* < 0.001; Fig. 7).

## Discussion

Anchialine caves represent an understudied environment, especially at the microbiological level [37]. In this context, our study provides an overview of the microbial life (microeukaryotic and prokaryotic community) in the water column throughout the halocline of this enigmatic karstic environment in the Mediterranean region. Investigated caves are located on nearby islands and connected to the same marine basin. The surface area of caves is influenced by different anthropological or non-anthropological interventions (birds or bat nests, Roman amphorae). These specific inland environments characterized by a salinity-stratified water column and saltwater exchange with the sea, specific physical and chemical parameters, depth, and isolated position represent the appropriate study sites for allopatric speciation processes. The observed microbial biodiversity is comparable with different cave types in other environments [38]. The conducted CIA resulted in a strong statistically significant correlation between microeukaryotic and prokaryotic communities in the different caves (Fig. 7). This site-specific adaptation is a very rare case in marine environments [39]. Future research will analyze the surrounding marine basin and compare it with the cave’s microbial community at different time scales. Another interesting point of our results is the low percentage of shared OTUs between the caves (only 2% in the prokaryotic and 8.1% in the microeukaryotic community) and the lack of the distance-decay relationships, which can be explained by only one sampling date per cave. OTU analysis on high taxonomic levels demonstrated that the taxon composition shifted markedly by anchialine cave and sampling depth both in the microeukaryotic and prokaryotic communities. The difference can be governed by some physical or chemical data that we have not measured or by the mass effect and dispersal theory, but all this has to be studied in more depth.

Only in the BP cave, the richness and the Shannon index of the microeukaryotic community had not followed a similar pattern. This cave is the deepest among other sampled caves and the most branched with speleothems in the submerged part. The highest microeukaryotic richness was detected in the area below the halocline in GKV and ZVP cave, while the lowest in the area above and below the halocline in the VG cave. Alveolata and Stramenopiles were the most diverse and abundant groups, as observed in other ecosystems [40, 41]. A decrease in Alveolata with depth was detected in two caves (BP and GKV), where an increase of Fungi with depth was recorded. Commonly found in marine and brackish water, ciliates affiliated to the genus *Mesodinium* were the major contributor to the microeukaryotic community above the halocline in the VG cave [42]. In and below the halocline, Alveolata was represented with the highest relative abundance of OTUs affiliated to the apicomplexan parasite *Besnoitia*. Compared to the VG cave, different Alveolata genera (*Oxyrrhis*) dominated the area with the lowest salinity concentration in the BP cave. Despite being known as globally distributed euryhaline and eurythermal genus with prevalence in intertidal pools and estuaries, it was reported that reduced salinity, caused by freshwater inflow, may stimulate blooms of this genus [43]

In the GKV cave, the geographically most distant cave that during the Roman time was used as a freshwater source, Alveolata were highly dominated by Ciliophora. The area above the halocline was dominated by the subclass Scuticociliatia. This subclass gathers free-living ciliates in fresh, brackish, and marine water together with opportunistic or facultative parasitic ciliates of aquatic animals [44]. Parasitic ciliate *Uronema* (Scuticociliatia) produces proteases responsible for the digestion of the host’s tissues and proteins responsible for the high mortality rates of fish [45]. However, fishes were not recorded in any of the investigated cave. The lowest OTU richness was recorded in the halocline, where the non-photosynthetic phagotrophic chrysomonads *Paraphysomonas* contributed with a high relative abundance (83.5%). Usually known as important feeders on bacteria, *Paraphysomonas* can be found as a freely swimming cell and also occurring attached to bacterial mats or other surfaces [46].

Compared to other sampled caves, the highest microeukaryotic diversity based on the higher taxon groups was established in the ZVP cave, the shallowest sampled cave, with the dominance of Stramenopiles, Alveolata, Fungi, Viridiplantae, Heterolobosea, and Rhizaria in the surface area. The relative abundance of Stramenopiles and Fungi has followed the decrease by the depth within the salinity gradient, whereas the relative abundance of Alveolata increased, ranging from 30.6 to 71.0%. Above the halocline, OTUs affiliated to Chrysophyceae and Synurophyceae reached a total relative abundance of 40.5%, with the highest contribution of uncultured Chrysophyceae (32%) and *Poterioochromonas* (4.6%) [47]. The fungal community within the salinity gradient shifted the dominance of genera from *Malassezia* (Basidiomycota) to *Verrucoconiothyrium* (Ascomycota). Ciliates (scuticocilate *Protocyclidium* and Holotrichous ciliate *Prorodon*) dominated in the low-nutrient conditions in and below the halocline. Fungi were numerous in all layers with the highest abundance (69.1%) in the hypoxic marine-like area, dominated by genera *Malassezia*, *Cladosporium*, and *Pseudobensingtonia*. Species of these genera often have pathogenic or saprophytic lifestyles, e.g., *Malassezia* is a lipid-dependent basidiomycetous yeast accounting for the majority of the eukaryotic diversity in deep-sea subsurface sediments [48].

The prokaryotic community was partially dominated in all the investigated caves by Gammaproteobacteria, and it was not possible to identify a partially similar pattern in richness and Shannon index as was in the microeukaryotic community. In the shallowest cave (ZVP), where the sunlight enters into the surface layers of the cave, the highest richness was detected. Although this cave was not the richest in nutrients, this is the only cave where the influence of the light could have an impact on the community. In the VG cave, the abundance of Thaumarchaeota, Bacteroidetes, Gemmatimonadetes, and Nitrospirae decreased with the increasing salinity gradient, while the abundance of Gammaproteobacteria, Firmicutes, and Actinobacteria was greatest below the halocline. The ammonia-oxidizing archaea Nitrososphaeria, which relies solely on the energy generated from the oxidation of ammonia, was recorded in high abundance above (29%) and the area of halocline (12.8%), corresponding with the highest concentration of ammonium with organic nitrogen (4.3 mg L^−1^) [49]. Bacteroidetes, together with a polyphosphate accumulating Gemmatimonadaceae [50], were also abundant in the surface area, contributing to chemoheterotrophy. The lowest DO concentration (1.9 mg L^−1^) was measured below the halocline, where the Gammaproteobacteria, Firmicutes, and Actinobacteria had the highest relative abundance. The common kestrel nests and bats were detected in this cave that could have a possible contribution to the source of pathogenic bacterial strains below the halocline. The only genus contributing to the prokaryotic community below the halocline that is not corroborated as pathogenic was *Lactobacillus*.

In the prokaryotic community of the BP cave, the most prominent archaeal lineage was Thaumarchaeota, with a similar contribution to the relative abundance and decreasing by the increasing salinity as in the VG cave. The ammonia-oxidizing archaea,* Nitrosopumilus* and *Nitrosoarchaeum*, reached a relative abundance of 25.2% in the surface area despite the low concentration of ammonium with organic nitrogen (0.218 mg L^−1^). Bacteroidetes, including the strictly aerobic and chemoorganotrophic family Cryomorphaceae and *Algoriphagus* (Cyclobacteriaceae), were abundant in the area above the halocline. Although previous studies showed their prevalence in the productive ocean and coastal regions, no specific associations with organic matter of Cryomorphaceae are known [51]. The transition of genera in the salinity gradient was demonstrated by the relative abundance of Actinobacteria and Gammaproteobacteria. In the surface area, Candidatus *Aquilina* and RS62 marine group dominated, while in the marine-like area, *Rhodococcus* and order Pseudomonadales. The highest OTU richness of the prokaryotic community in this cave was determined in the hypoxic halocline. This could be correlated to the higher bacterial activity in this layer [52]. Below the halocline recorded community was characterized as pathogenic with the majority of the genera related to *Rhodococcus*, *Sphingomonas*, *Arcobacter*, *Lawsonella*, and *Staphylococcus*. The source of this pathogenic bacteria remains unclear.

The main prokaryotic groups detected in the surface area of the GKV cave were Gammaproteobacteria, Alphaproteobacteria, and Bacteroidetes. Ubiquitous gram-negative and non-fermenting coccobacilli *Acinetobacter* (11.5%) had the highest relative abundance among other Gammaproteobacteria together with ubiquitous gram-negative and aerobic or facultatively anaerobic *Myroides* (9.1%) from phyla Bacteroidetes. *Acinetobacter* species are widely distributed in nature and their growth may be enhanced by the contaminated environment such as hydrocarbon-contaminated areas, activated sludge, sewage [53], whereas relatively little is known of pathogenic genus *Myroides* with proven high multi-drug resistance [54]. The lowest OTU richness of the prokaryotic community in GKV cave was recorded in the area of halocline with the highest abundance of Actinobacteria and Rhodobacteraceae. Marine actinobacterial lineage PeM15 (39.1%) is identified in various habitats from aerobic to anaerobic environments and is very sensitive to nutrient enrichment [55]. Heterotrophic Marine Group II (Thermoplasmata) has reached the highest relative abundance above and below the halocline known to reside mostly in the photic zone with unique organic carbon degradation pattern [56].

The highest concentration of ammonium with organic nitrogen and TOC was measured in the surface area of the ZVP cave, potentially contributing to the development of the prokaryotic community. The relative abundance of Gammaproteobacteria, Alphaproteobacteria, Bacteroidetes, and Actinobacteria decreased with the increasing salinity gradient, while Epsilonbacteraeota, Omnitrophicaeota, and Marinimicrobia (SAR406 clade) were most abundant in the marine-like layer. The genus *Limnohabitans* had the greatest relative abundance (18.8%) compared to other detected Betaproteobacteriales. This genus is characterized by a high growth rate and metabolic flexibility with a notably tight relationship to algae-derived organic substances [57]. Decreased DO and low nutrient concentration contributed to the diversity of the prokaryotic community in and below the halocline, with the highest OTU richness recorded below the halocline. Genera associated with chemically distinct environments enriched with sulfur compounds were detected in the halocline (*Sulfurimonas* (Epsilonbacteraeota), Ectothiorhodospiraceae (Gammaproteobacteria), and *Desulfatiglans* (Deltaproteobacteria)). In depth with the highest salinity and lowest DO, archaeal phylum Epsilonbacteraeota, Omnitrophicaeota, and Gammaproteobacteria were detected [58, 59].

Our study identifies specific transitional boundaries for microeukaryotic and prokaryotic communities in the salinity gradient of anchialine caves. These transition boundaries are not restricted and it remains unclear if and to what extent the microeukaryotic and prokaryotic communities respond to the salinity gradient. Each anchialine cave had a unique microeukaryotic and prokaryotic community, indicating that cave niches play an important role in determining cave microbial diversity. This result also confirms the highly endemic character of anchialine environments and targeted studies should therefore be carried out to reveal the extent of the diversity and the ecological roles of the microeukaryotic and prokaryotic communities.

## Supplementary Information

Below is the link to the electronic supplementary material.

Supplementary file1 (DOCX 407 KB)

## Data Availability

Raw demultiplexed reads were deposited at the ENA’s Sequence Read Archive and are publicly available under project number PRJEB43761.

## Code Availability

A simplified version of the R script is available online (https://github.com/kkajan/anchialine-miceco).
