# Supplementary material for: Microeukaryotic and Prokaryotic Diversity of Anchialine Caves from Eastern Adriatic Sea Islands
Source: Microb Ecol. 2021 Apr 26;83(2):257–70. doi: 10.1007/s00248-021-01760-5 (PMC8891109; doi:10.1007/s00248-021-01760-5)
Supplement: Supplementary file 1 — Supplementary file1 (DOCX 407 KB) [file 248_2021_1760_MOESM1_ESM.docx]

Supplementary Material


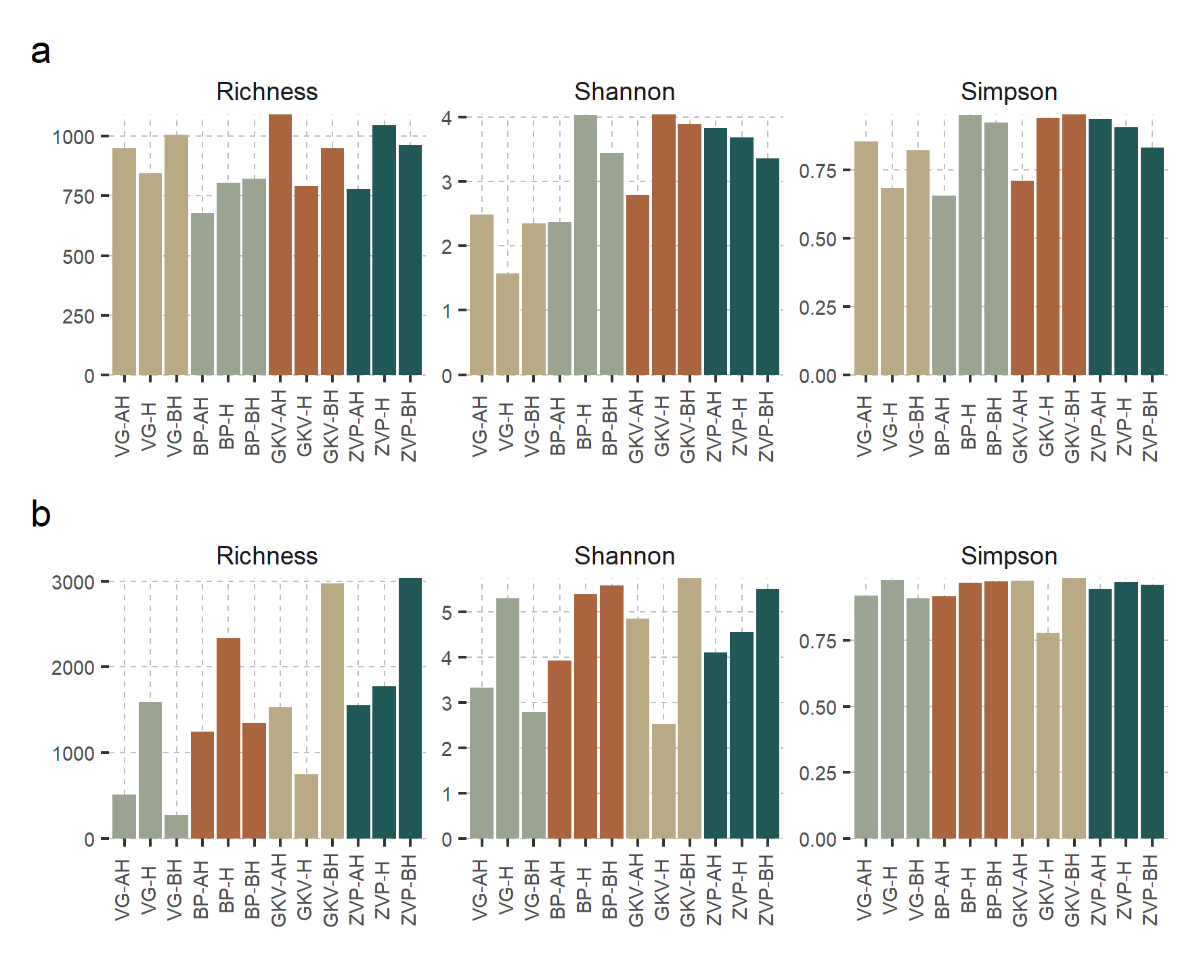


**Fig. S1** Alpha diversity of microeukaryotic (**a**) and prokaryotic community (**b**). Sample name abbreviations refer to the area of a sampling point: above halocline (AH), in the halocline (H) and below halocline (BH)

**Table S1** Values of measured chemical parameters in anchialine caves

| Sample | TN (mg L^-1^) | Nitrate (mg L^-1^) | Nitrite (mg L^-1^) | Ammonium+Norg (mg L^-1^) | orto-P (mg L^-1^) | TOC (mg L^-1^) |
| --- | --- | --- | --- | --- | --- | --- |
| VG-AH | 7 | 2.7 | 0.003 | 4.297 | 0.044 | 0.71 |
| VG-H | 1.3 | 0.41 | 0.001 | 0.89 | 0.033 | 0.49 |
| VG-BH | 0.15 | 0.12 | 0.001 | 0.03 | 0.011 | 0.4 |
| BP-AH | 0.39 | 0.17 | 0.002 | 0.218 | 0.017 | 1 |
| BP-H | 0.15 | 0.015 | 0.001 | 0.15 | 0.019 | 0.67 |
| BP-BH | 0.2 | 0.11 | 0.001 | 0.089 | 0.019 | 0.47 |
| GKV-AH | 1.4 | 0.04 | 0.001 | 1.36 | 0.009 | 1.2 |
| GKV-H | 0.15 | 0.09 | 0.001 | 0.06 | 0.006 | 1.1 |
| GKV-BH | 0.08 | 0.03 | 0.001 | 0.05 | 0.011 | 0.49 |
| ZVP-AH | 3 | 0.78 | 0.007 | 2.213 | 0.006 | 1.3 |
| ZVP-H | 0.54 | 0.02 | 0.002 | 0.518 | 0.033 | 1.2 |
| ZVP-BH | 0.36 | 0.015 | 0.001 | 0.36 | 0.032 | 0.87 |
